# Current induced polycrystalline-to-crystalline transformation in vanadium dioxide nanowires

**DOI:** 10.1038/srep37296

**Published:** 2016-11-28

**Authors:** Junho Jeong, Zheng Yong, Arash Joushaghani, Alexander Tsukernik, Suzanne Paradis, David Alain, Joyce K. S. Poon

**Affiliations:** 1 Department of Electrical and Computer Engineering, University of Toronto, Toronto, Ontario, M5S 3G4, Canada; 2 Toronto Nanofabrication Centre, University of Toronto, Toronto, Ontario, M5S 3G4, Canada; 3 Defence Research and Development Canada - Valcartier, Quebec, Quebec G3J 1X5, Canada

## Abstract

Vanadium dioxide (VO_2_) exhibits a reversible insulator-metal phase transition that is of significant interest in energy-efficient nanoelectronic and nanophotonic devices. In these applications, crystalline materials are usually preferred for their superior electrical transport characteristics as well as spatial homogeneity and low surface roughness over the device area for reduced scattering. Here, we show applied electrical currents can induce a permanent reconfiguration of polycrystalline VO_2_ nanowires into crystalline nanowires, resulting in a dramatically reduced hysteresis across the phase transition and reduced resistivity. Low currents below 3 mA were sufficient to cause the local temperature in the VO_2_ to reach about 1780 K to activate the irreversible polycrystalline-to-crystalline transformation. The crystallinity was confirmed by electron microscopy and diffraction analyses. This simple yet localized post-processing of insulator-metal phase transition materials may enable new methods of studying and fabricating nanoscale structures and devices formed from these materials.

Materials capable of insulator-metal phase transitions (IMTs) enable nanoelectronic and nanophotonic devices, such as transistors^1^,^2^,^3^, memristors^4^,^5^,^6^, solid-state memory^7^,^8^,^9^, and optical modulators^10^,^11^, with extraordinarily steep turn on characteristics and high on-off ratios that can dramatically improve the energy-efficiency of computing and communications. An example is the correlated electron material, vanadium dioxide (VO_2_), which exhibits an IMT that can be induced by temperature, current, voltage, ionic gating, and electromagnetic radiation^12^,^13^,^14^,^15^,^16^,^17^,^18^. In many device applications, epitaxial quality crystalline films are often preferred for improved electrical transport characteristics and spatial homogeneity. Until now, the formation of crystalline VO_2_ films by methods such as pulsed laser deposition^17^, chemical vapor deposition^19^, sputtering^20^, and vapour transport method^21^,^22^ has required the seeding from a suitably lattice-matched crystalline substrate, most often Al_2_O_3_ or TiO_2_^19^,^20^,^22^,^23^. Here, we demonstrate that nanowires etched from polycrystalline VO_2_ films sputtered on thermally grown silica (SiO_2_) on silicon (Si) can be permanently transformed to crystalline nanowires via the application of electrical currents. Thermal modelling reveals that the applied current causes heating localized to the VO_2_ nanowire, which likely led to the rearrangement of grain boundaries in VO_2_. Our discovery is a new way to selectively and rapidly post-process nanostructures of polycrystalline VO_2_, and potentially other phase transition materials, deposited on amorphous substrates into a crystalline form.

To crystallize VO_2_ on amorphous substrates, we drew inspiration from the recrystallization of metals and amorphous Si in which thermal annealing is often used to rearrange existing grain boundaries and promote the growth in size of the newly nucleated grains^24^,^25^,^26^. We postulated that if sufficient localized heating is applied to polycrystalline VO_2_ micro/nanostructures, VO_2_ could crystallize upon cooling without the need to heat the entire substrate. Here in our manuscript we define the term “polycrystalline” to be a form when the grain size in the film is less than 100 nm in width and the “crystalline” form is when the grain boundaries are no longer observable through electron microscopy and diffraction analyses. Moreover, if the VO_2_ micro/nanostructure is smaller than the final grain size, the resultant structure would effectively be “single crystalline”. A way to deliver heating that is local and specific to the VO_2_ micro/nanostructure is by applying an electrical current. For sufficiently small cross-section areas, large current densities, hence sufficient heating, may be achieved at low (<3 mA) applied currents and the crystallization process can be extremely rapid. This process is in contrast to the typical thermal annealing of VO_2_, in which the film is heated gradually and uniformly to reduce V_2_O_5_ into VO_2_ or create oxygen vacancies^27^,^28^,^29^.

## Results

### Two terminal device and electrical characterization

**Figure 1:**
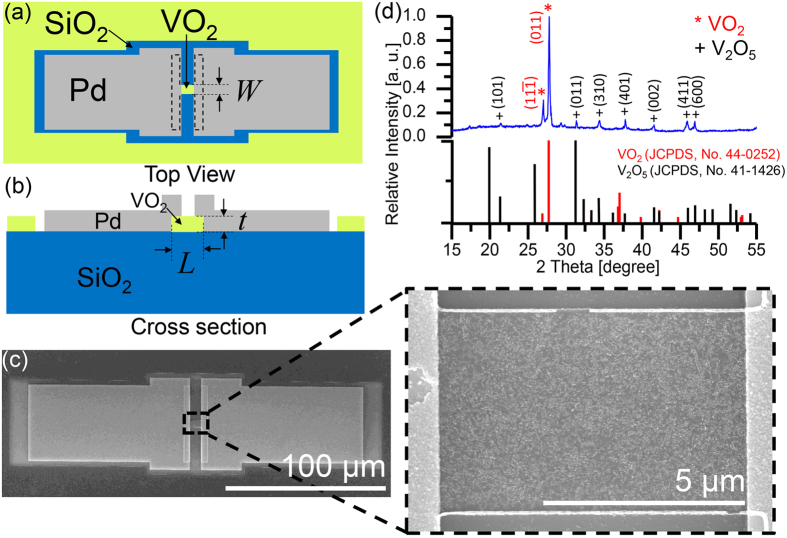
The fabricated device and the as-sputtered VO_2_ film. The (**a**) top and (**b**) cross-section view schematics of a VO_2_ device with Pd contacts. (**c**) SEM images of a device at two magnifications. (**d**) X-ray diffraction pattern of the starting VO_2_ film before crystallization.

Figure 1(a) and (b) show the top and cross-section view schematics of the two terminal VO_2_ nanowire devices fabricated to demonstrate and investigate this phenomenon. A 120 nm thick VO_2_ film was deposited onto a 2 *μ*m thick thermally grown SiO_2_ on a Si substrate using radio-frequency (RF) magnetron sputtering^11^. The deposition results in polycrystalline films. Then we fabricated sets of VO_2_ nanowires with varying widths and lengths using electron beam lithography and reactive ion etching. The two lateral electrodes were formed by electron-beam lithography and lift-off of a 100 nm thick palladium (Pd) film. The contacts were separated by a gap of length *L* varying between 0.75 *μ*m and 7.5 *μ*m, and the VO_2_ wire width, *W*, varied between 0.40 *μ*m and 50 *μ*m. We chose to use the two-contact geometry, rather than the four-point probe geometry in ref. 30, because it allows for the straightforward measurement of the voltage vs. current (*VI*) characteristics before and after the crystallization using the same devices. Details of the fabrication are described in the Methods section.

To ensure that the contact resistance between the Pd contact pads and VO_2_ film would not have a significant contribution to the total measured resistance, the surface area of the VO_2_ film under each Pd contact was kept to a large area of 4 *μ*m × 50 *μ*m. This surface area was identical for all the devices such that their contact resistances would be nominally identical. The H shaped VO_2_ region is indicated by the long dashed line in Fig. 1(a). Figure 1(c) shows a scanning electron microscope (SEM) images of a device with *L* = 7.5 *μ*m and *W* = 5.0 *μ*m. The X-ray diffraction (XRD) scan of the VO_2_ film in Fig. 1(d) shows that the film was dominantly VO_2_ but also had some contribution of V_2_O_5_. The calculated weight percentage of VO_2_ and V_2_O_5_ was 68.9 *wt*.% and 31.1 *wt*.%, respectively. The broad low amplitude peak between 15 °2*θ* and 47 °2*θ* with a maximum around 22 °2*θ* in the scan is due to the amorphous SiO_2_ under the VO_2_ film.

**Figure 2:**
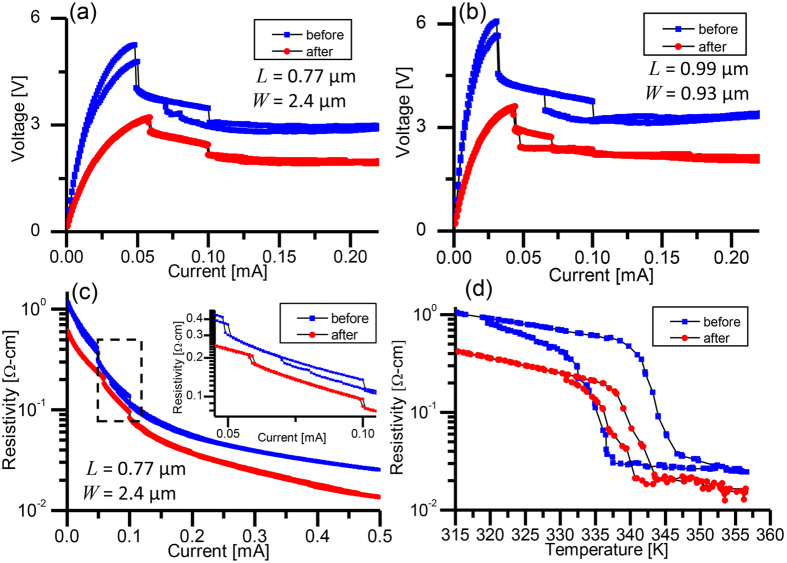
*VI* and resistivity measurements before and after crystallization. *VI* measurements for (**a**) *L* = 0.77 *μ*m and *W* = 2.4 *μ*m and (**b**) *L* = 0.99 *μ*m and *W* = 0.93 *μ*m. (**c**) The resistivity vs. current corresponding to (**a**) and the inset is the magnified region in dashed lines. (**d**) The resistivity vs. temperature for the “before” and “after” states showing the reduced hysteresis and resistivity in the “after” state.

The fabricated devices were measured by contacting tungsten probes to the two Pd pads. Using a precision sourcemeter with a 1 kΩ resistor in series, current was applied to the devices. This resistor protected the device from being overdriven during the IMT. First, we obtained the “before” *VI* relations of the polycrystalline VO_2_ by sweeping the applied current between 0 mA and 0.3 mA with 1 *μ*A increments, which is applied for 2.7 *μ*s. We ensure that the devices undergo the nominal current-induced two-step phase transition in VO_2_^31^,^32^ before increasing the maximum current limit. A swept voltage measurement would result in only a single step transition^12^,^15^,^33^. Two example *VI* plots are shown in blue in Fig. 2(a) and (b) for VO_2_ nanowires with two different dimensions. The “before” *VI* curve has a large hysteresis in the second step due to the Joule heating assisted phase transition^15^. The width of the hysteresis, as well as the current and voltage of the first and second transition depends on the dimension of the VO_2_ wires^32^.

After the “before” measurements, for each sample, we increased the applied current beyond 0.3 mA in increments of 0.2 mA until we observed a rapid, abrupt drop in the voltage at a critical current, *I*_*C*_ (Supplementary Figure S1). The voltage drop occurred on time-scales shorter than the data acquisition time in our measurement setup of about 2.7 *μ*s. After the voltage drop, the current was ramped down. The change in the resultant *VI* was permanent, and we obtained the “after” *VI* relation by sweeping the applied current between 0 mA and 0.3 mA. The red lines in Fig. 2(a) and (b) show the “after” *VI* curves of the VO_2_ wires after applying *I*_*C*_ of 2.3 mA and 0.68 mA, respectively. As seen in both Fig. 2(a) and (b), the transition voltage at the first step decreased by approximately 50% and the hysteresis in the second step, from the thermal part of the phase transition^11^, was significantly reduced. The hysteresis in Fig. 2(a) has nearly collapsed completely. As discussed in the Supplementary Information, we found that the contact resistances in the VO_2_ insulating and metallic phases, *R*_*con,i*_ and *R*_*con,m*_, in the “before” and “after” states were within the same order of magnitude (“before”: *R*_*con,i*_ = 62.2 ± 9.7 kΩ, *R*_*con,m*_ = 580 ± 53 Ω; “after”: *R*_*con,i*_ = 54.6 ± 7.1 kΩ, *R*_*con,m*_ = 336 ± 49 Ω). This means the change in the *VI* characteristics was not due to the contacts but rather the VO_2_ wire. Furthermore, this suggests the crystallinity of the VO_2_ film under the Pd contacts experienced minimal change.

Figure 2(c) compares the “before” and “after” resistivity, *ρ*, as a function of current for the device of Fig. 2(a) (details on the resistivity calculations are described in the Supplementary Information). The “after” plot shows that the hysteresis has been dramatically reduced and large resistivity discontinuities are absent. The almost continuous resistivity vs. current characteristics and low hysteresis width are useful for devices requiring continuous control over resistance and would suggest improved switching speeds. The reduction in the resistivity change is also observed in the *ρ* vs. temperature plot in Fig. 2(d). The resistivity of the “after” state is about 2.5 times lower than the “before” state for both the insulator and metallic phases of the VO_2_. This is consistent with an increased carrier scattering time or mean free path in the material by at least 2.5 times. However, the overall change in resistivity across the phase transition was reduced. The ratio between the metallic and insulator phase resistivity in the “before” case is 2.3 × 10^−2^, and the resistivity ratio in the “after” case is 3.6 × 10^−2^. The “before” and “after” phase transition temperatures were about the same at approximately 343 K, but in the “after” case, the hysteresis width was reduced to about 3 K, identical to that of single crystal VO_2_^19^,^20^,^23^,^34^. Furthermore, all the *VI* and *ρ* vs. temperature measurements were repeatable in both the “before” and “after” case. Device failure occurred when a current beyond *I*_*C*_ was injected into the device.

Due to the small size of the wires and the device geometry, it is difficult to directly determine the material composition, stochiometry, or the vanadium valence state in the “after” state using standard techniques such as XRD or X-ray photoelectron spectroscopy (XPS). However, the “after” state should be dominantly VO_2_ since the phase transition temperature in the “before” and “after” cases are similar (Fig. 2(d)). It is unlikely that the VO_2_ wires were converted to other vanadium oxides, such as VO, V_2_O_3_, or V_2_O_5_, which have drastically different phase transition temperatures compared to VO_2_ (for example, the transition temperature of V_2_O_3_ and V_2_O_5_ are around 150 K and 553 K, respectively^35^,^36^). The reduction in the resistivity change in the “after” state may be due to the small quantity of V_2_O_5_ (Fig. 1(d)) or changes in the strain in the VO_2_ wires^37^.

### Electron microscopy and diffraction analyses

**Figure 3:**
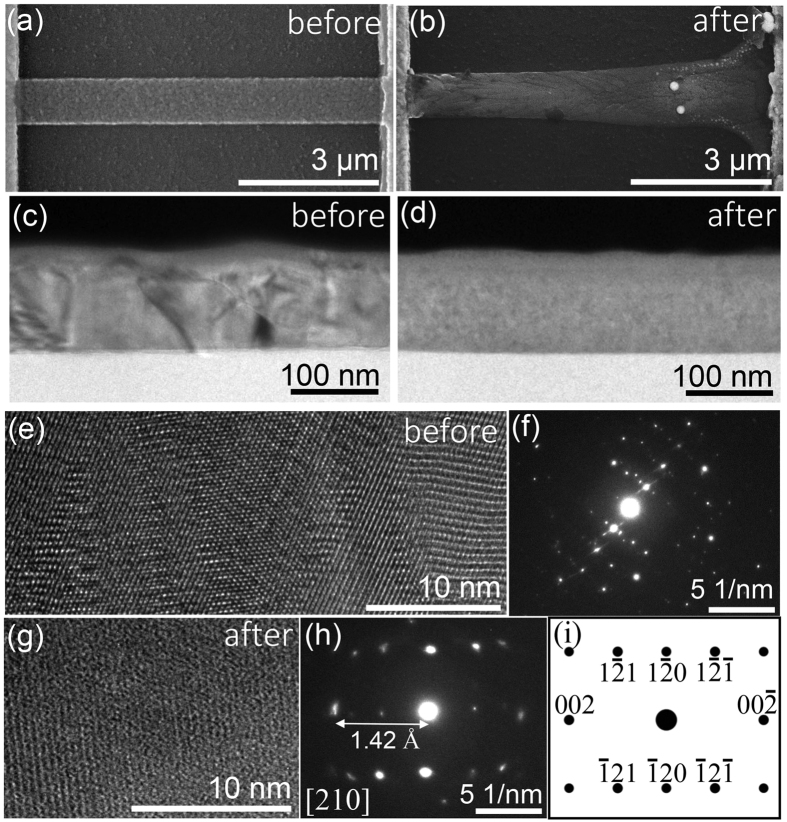
Electron microscopy images and diffraction patterns before and after the crystallization. Top view SEM images of the VO_2_ nanowire device with *L* = 7.5 *μ*m and *W* = 0.91 *μ*m (**a**) before and (**b**) after the crystallization. XTEM images of a VO_2_ nanowire (**c**) before and (**d**) after crystallization showing the absence of grains in the “after” state. (**e**) is the high-resolution TEM and (**f**) the SAED pattern corresponding to the “before” state in (**c**). The polycrystallinity is evident in the non-uniform plane orientations in (**e**) and randomness of the diffraction pattern in (**f**). (**g**) is the high-resolution TEM and (**h**) the SAED pattern corresponding to the “after” state in (**d**) taken in the VO_2_ metallic (rutile) phase. The atomic arrangement is more periodic and the diffraction pattern exhibits clear symmetries that can be matched to the (**i**) computed diffraction pattern of VO_2_ with a rutile crystal structure at view direction [210] using the lattice parameters from ref. 38.

The “after” state of the devices was found to be due a permanent structural transformation of the polycrystalline VO_2_ into crystalline VO_2_. Figure 3 shows SEM, cross-sectional transmission electron microscope (XTEM) and selected area electron diffraction (SAED) images of the VO_2_ nanowires in the “before” and “after” states. Figure 3(a) and (b) show the “before” and “after”, respectively, top-view SEM images of the device with *L* = 7.5 *μ*m and *W* = 0.91 *μ*m. The top surface of the VO_2_ in Fig. 3(a) is rough with visible grain boundaries. From the XTEM image of the VO_2_ film in the “before” state in Fig. 3(c), the grain sizes are estimated to be about 75 ± 25 nm in width. In Fig. 3(b), the VO_2_ surface is smooth with no visible grain boundaries. The expanded width on the right end of the wire is suggestive of material reflow. The XTEM of the “after” state in Fig. 3(d) also shows the absence of grain boundaries. Because only a small region could be imaged, grain boundaries could exist along the VO_2_ wire width and length and we were not able to confirm whether the wire was entirely single crystal. For devices with *W* > 5 *μ*m, only a narrow region in the VO_2_ wire appeared smooth, suggesting that the current was concentrated through a filament that had a minimum resistance between the two contact pads (Supplementary Figure S3). The width of the narrow, smooth region is consistent with our previous measurements of the width of thermal filaments in ref. 32.

Figure 3(e) and (f) respectively show high-resolution XTEM and SAED images of the VO_2_ in a device at room temperature. The images were taken over a sample diameter of 150 nm. Numerous crystal orientations are evident in Fig. 3(e) and the randomness of the diffraction pattern in Fig. 3(f) confirm the polycrystallinity of the VO_2_. In contrast, the high-resolution XTEM and SAED images of VO_2_ in Fig. 3(g) and (h), respectively, clearly shows crystalline characteristics. These images have been taken at the metallic phase of the VO_2_ at 80 °C and were taken from a device different from that in Fig. 3(e) and (f) due to the need for sample preparation (Supplementary Figure S4). In Fig. 3(g), the atomic arrangement is spatially regular without any grain boundaries, and the clear diffraction pattern exhibiting symmetries in Fig. 3(h) further suggests the VO_2_ is crystalline. Using the SingleCrystal software package, we matched the observed diffraction pattern with the expected diffraction pattern using the lattice parameters of VO_2_ in the metallic phase (rutile structure) from previous studies^38^. The calculated diffraction pattern is shown in Fig. 3(i). A challenge in matching the computed diffraction pattern with the measurement was the unknown orientation of the VO_2_ sample relative to the incident electron beam. To determine the incident direction of the beam, in Fig. 3(h), we first identified a separation of 1.42 Å between the center of the diffraction pattern and the nearest diffraction spot in the horizontal direction. This distance is similar to half of the *c*-axis lattice constant of VO_2_ (*c* = 2.85 Å), so we identified the diffraction spot to be from the (002) plane and varied the electron beam incident direction until the rest of the diffraction pattern was matched. A confirmation of the fitting is that with the correct view direction of the diffraction pattern, [210], the simulated diffraction pattern aligns with the SAED pattern. The faint spots in the diffraction pattern that do not match with the VO_2_ are likely due to the residual V_2_O_5_. The complete fittings of the diffraction patterns for both the monoclinic and rutile phases and the contribution from V_2_O_5_ are included in the Supplementary Information.

### Thermal modelling

**Figure 4:**
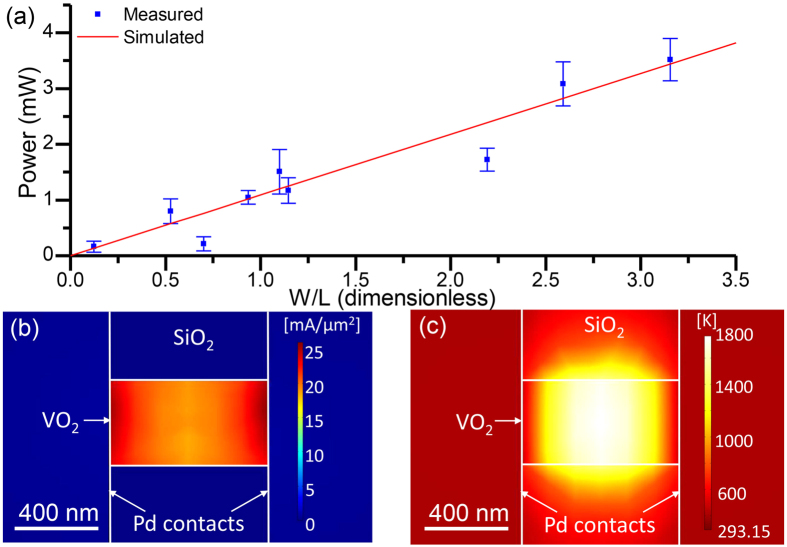
Thermal modeling of the crystallization. (**a**) The measured dissipated power at the critical current compared to the power required to heat the VO_2_ nanowires by Δ*T* = 1470 K calculated using (**a**) 1D Fourier model for devices with varying *W/L*. The result of a thermal transport simulation (COMSOL Multiphysics) showing (**b**) the current density and (**c**) temperature distribution for VO_2_ wire of dimensions *L* = 0.78 *μ*m and *W* = 0.41 *μ*m at the critical current, *I*_*C*_.

Through measurements of devices with varying *W* and *L* combined with heat transport simulations, we can ascribe the observed polycrystalline-to-crystalline transformation to a thermally activated process, akin to recrystallization in metals and amorphous semiconductors^24^,^25^,^26^. Applying Fourier’s law of heat conduction (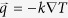, where 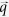 is the heat flow, *k* is the thermal conductivity, and *T* is the temperature) to the VO_2_ nanowire, in the one-dimensional steady-state, if 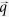 is taken to be the dissipated power (*P*) over cross-section area (*Wt*), then


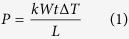


where Δ*T* is the change in temperature. Therefore, the dissipated power is expected to be linearly proportional to the geometric parameter, *W/L*. In our experiment, 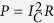, with *R* = *R*_*tot*_ − 2*R*_*con,m*_ where *R*_*tot*_ is the total measured resistance of the device. Figure 4(a) shows the measured dissipated electrical power, *P*, at the critical current for the structural change is indeed a linear function of *W/L*. Using the thermal conductivity of VO_2_ in ref. 39, the measured data is fitted to Equation 1 with Δ*T* = 1470 K as shown in Fig. 4(a) in red. This result suggests that the local temperature of the VO_2_ wire was around 1770 K for the polycrystalline-to-crystalline transformation to occur. This temperature is about 80% of the melting point of VO_2_ of 2240 K, which is consistent with the requirements of recrystallization processes^24^,^25^. For example, amorphous Si can be recrystallized at around 773–913 K even though the melting point of Si is 1687 K^24^,^25^. In recrystallization, the energy required for nucleation and grain growth responsible for the change in crystallinity is less than the energy required for melting^25^,^26^.

To obtain a more accurate estimate of the temperature for the polycrystalline-to-crystalline transformation and to confirm whether such temperatures could have been generated in our experiment, we simulated the temperature profile in VO_2_ nanowires, including the Pd contacts and substrate, at the critical current. The calculations were performed using Joule heating module of COMSOL Multiphysics a stationary study in three dimensions using a non-uniform mesh (minimum mesh size of 5 nm) for different VO_2_ wire dimensions. The top surfaces of the structure are modelled as convective cooling surfaces allowing thermal transport into the air. The side and bottom boundaries were set to room temperature and were sufficiently far away from the VO_2_ wire such that they did not influence the results of the heat transport in the wire. The heat capacity of VO_2_ was modelled as ref. 40 and the thermal conductivity values were taken from ref. 39. In the thermal simulations, the applied voltage across the contact pads is varied to match the current density through the VO_2_ nanowire with the experimental critical current density required for crystallization. Figure 4(b) and (c) show the matched simulated current density and corresponding temperature profile, respectively, for the VO_2_ wire with *L* = 0.78 *μ*m and W = 0.41 *μ*m. The current density is the highest near the interface between the Pd contacts and VO_2_ wire. The centre of the VO_2_ wire reaches the highest temperature of 1780 K, similar to the value extrapolated from the one dimensional Fourier model and Fig. 4(a) of 1770 K. Different wire dimensions resulted in similar maximum temperature values to within 10%. Interestingly, the highest temperature region is localized to the centre of the nanowire, which may explain why the contact resistance was unchanged in the polycrystalline-to-crystalline transformation as the temperature may not have been sufficiently high at the Pd contacts for VO_2_ to crystallize. Moreover, at these high temperatures it is unlikely that VO_2_ will further oxidize with the oxygen in the air to form V_2_O_5_. This is because the boiling temperature of V_2_O_5_ is 1750 K^41^ and at around 1770 K it will most likely thermally decompose.

## Discussion

In summary, we have demonstrated the transformation of polycrystalline VO_2_ into crystalline VO_2_ in nanowire geometries by injecting electrical currents of the order of 1 mA to reach local temperatures near 1780 K. The reconfiguration is likely through a process akin to recrystallization in metals and semiconductors by thermal annealing, with a key difference that the transformation here was rapid, occurring within microseconds. The final crystal orientation of the VO_2_ nanowires was not controlled in these experiments, but it may be possible to induce a preferential orientation by introducing strain or patterning the substrate, for example. The crystallized devices exhibited reduced hysteresis across the phase transition and reduced resistivity, essential features for common electronic and photonic devices. Because polycrystalline VO_2_ films can be deposited at low temperatures (less than around 450–500 °C) on amorphous dielectrics or silicon, compatible with back-end of line complementary metal oxide semiconductor (CMOS) fabrication processing^17^,^28^, our findings open the potential for highly dense, three-dimensional integration of crystalline VO_2_ and other IMT materials with CMOS devices. Further, the simplicity of the current induced crystallization can broaden the investigation of crystalline VO_2_ and IMT nanostructures on unconventional substrates, including those that are lattice-mismatched, amorphous, or flexible.

## Methods

### VO_2_ deposition

The VO_2_ film was deposited using reactive RF magnetron sputtering of a 50 mm vanadium target (99.7% purity). A 4 cm × 4 cm piece of Si with a 2 *μ*m thick of thermally grown SiO_2_ was placed on a 150 mm diameter Si wafer, which served as the substrate holder. During the deposition, the substrate holder was held at 500 °C and the chamber was filled with high purity argon (Ar) and oxygen (O_2_) at flow rates of 80.0 sccm and 2.0 sccm, respectively at a total pressure of 10 mT. The RF power was ramped to 200 W and the O_2_ flow rate was closely monitored to keep the DC bias of the sample constant. The deposition rate was approximately 0.3 Å/s.

### Device Fabrication

The devices were fabricated using aligned electron-beam lithography, dry etching, and metal deposition. First, 20 *μ*m × 20 *μ*m tungsten alignment markers for the electron-beam lithography were formed onto the VO_2_ film. Then, the patterns for the VO_2_ nanowires were written using an electron-beam writer (Vistec EBPG 5000, at a dosage of 250 C/cm^2^ and a current of 500 pA) in a 450 nm thick layer of ZEP-520A resist. The chip was then developed in ZED-N50 for 60 s and then in methyl isobutyl ketone (MIBK) for 30 s. Prior to etching, the resist was baked for 4 min at 100 °C. The sample was then etched using an inductively coupled plasma reactive ion etcher (ICP-RIE). The sample was placed on an aluminum chuck and pre-heated on a hotplate to 80 °C prior to insertion into the ICP-RIE, so the VO_2_ was in its metallic phase during the etch. A gas mixture of Cl_2_ (6 sccm), H_2_ (6 sccm), and Ar (9 sccm) at a total pressure of 5 mT was used for the etch. At a RF power of 125 W and ICP power of 500 W, 50 s was required for a full etch of the VO_2_. After etching, the ZEP resist was removed by immersing the sample in ZDMAC for 10 min at 70 °C followed by 1 min of sonication in the same solution.With this fabrication process, the minimal feature size of the VO_2_ nanowire obtained was 400 nm in width and 750 nm in length. Finally, contact pads were formed on the VO_2_ nanowires by aligned electron-beam lithography (with ZEP-520A resist), thermal evaporation of a 3 nm thick chromium adhesion layer and a 100 nm thick Pd layer (deposition rate: 1.5 Å/s), and lift-off.

## Additional Information

**How to cite this article**: Jeong, J. *et al*. Current induced polycrystalline-to-crystalline transformation in vanadium dioxide nanowires. *Sci. Rep.* **6**, 37296; doi: 10.1038/srep37296 (2016).

**Publisher's note:** Springer Nature remains neutral with regard to jurisdictional claims in published maps and institutional affiliations.

## Supplementary Material

Supplementary Information
