# Supplementary material for: Current induced polycrystalline-to-crystalline transformation in vanadium dioxide nanowires
Source: Sci Rep. 2016 Nov 28;6:37296. doi: 10.1038/srep37296 (PMC5125010; doi:10.1038/srep37296)
Supplement: Supplementary Information [file srep37296-s1.pdf]

Supplementary Information

Current induced polycrystalline-to-crystalline transformation in vanadium dioxide nanowires

Junho Jeong[1,], Zheng Yong[1], Arash Joushaghani[1,†], Alex Tsukernik[2], Suzanne Paradis[3], David Alain[3], and Joyce K. S. Poon[1]*

[1]Department of Electrical and Computer Engineering, University of Toronto, 10 King's College Road, Toronto, Ontario, M5S 3G4, Canada.

[2]Toronto Nanofabrication Centre, University of Toronto, 6 King's College Road, Toronto, Ontario, M5S 3G4, Canada.

[3]Defence Research and Development Canada - Valcartier, 2459 de la Bravoure Road, Quebec, Quebec G3J 1X5, Canada.

*Correspondence to: dave.jeong@mail.utoronto.ca

†Present address: Intel, 2501 NW 229th Avenue, Hillsboro, OR 97124, USA.

This document includes:

- Description of experimental details
  - Critical Current
  - Determination of the Contact Resistance and VO_2 Resistivity
  - X-ray Diffraction and Electron Microscopy
  - Crystallization in Wide VO_2 Wires
- Table S1
  - **Table S1**. R_{con} for various VO_2 wire widths before crystallization in the insulator phase.
- Figures S1 – S4
  - **Figure S1.** *VI* plot showing the abrupt voltage drop at the critical current (I_C).
  - **Figure S2.** Extraction of the contact resistance. Total resistance vs. length of VO_2 devices before crystallization in the insulator phase.
  - **Figure S3.** Partial crystallization in wide VO_2 wires.
  - **Figure S4.** Measured and computed diffraction patterns.

Critical Current

Figure S1 (a) and (b) shows the abrupt irreversible voltage drop in the VO_2 wire at their respective critical currents, I_C, 2.3 mA and 0.7 mA. The *VI* plot show two different paths as the current is ramped up ("before" *VI*) and back down to 0 mA ("after" *VI*).

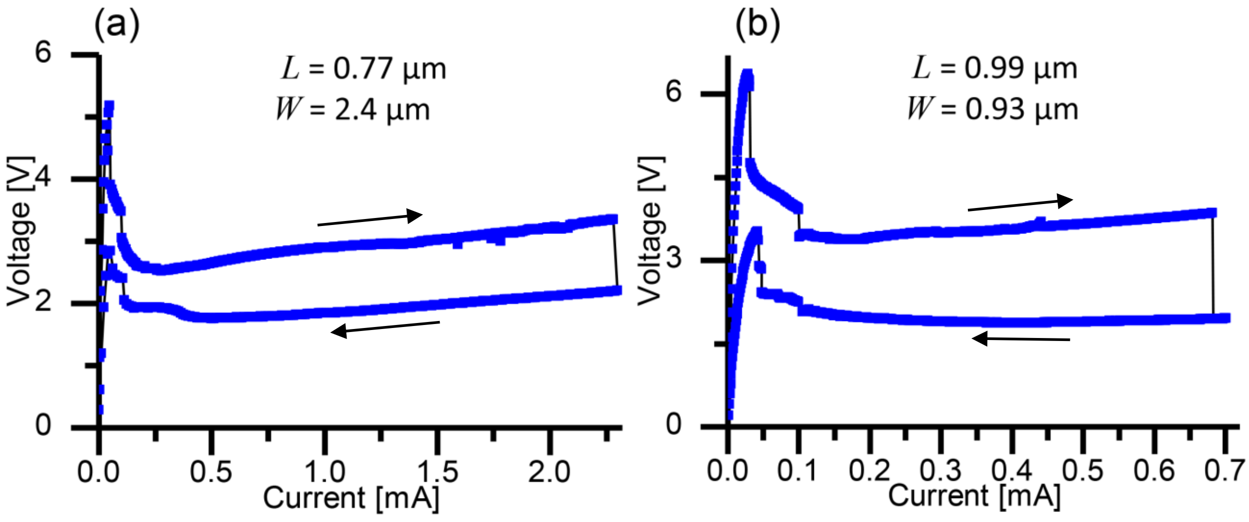

Figure S1. *VI* plot showing the abrupt voltage drop at the critical current (I_C). (a) VO_2 wire with dimensions L = 0.77 µm and W = 2.4 µm, and I_C = 2.3 mA, and (b) VO_2 wire with dimensions L = 0.99 µm and W = 0.93 µm, and I_C = 0.68 mA.

Determination of the Contact Resistance and VO_2 Resistivity

The contact resistance (R_{con}) of the 4 different scenarios (the insulator and metallic states before and after crystallization) are calculated using a modified transmission line method (TLM). Typical TLM involves a series of contact pads with varying lengths of separation on a single wire. In our case, we fabricated sets of wires with varying lengths and widths but kept the contact area between VO_2 and Pd constant, as explained in the main article. For each wire width, W, we plot the total resistance ($R_{total} = R_{VO2} + 2R_{con}$) vs. length as shown in Figure S2. The dimensions of $W1$ to $W6$ are listed in Table S1.

The total resistance was found by linearly fitting the first 5 data points from the ramp-up (0 mA to 0.3 mA) *VI* curve for the VO_2 insulating phase and the last 5 data points for the VO_2 metallic phase. In Figure S2, different wire widths lead to different slopes corresponding to R_{VO2}, and R_{con} should be roughly constant. To determine R_{con}, we averaged the intercept resistance values in Figure S2 and took the standard deviation to be the uncertainty in R_{con}. The results are summarized in Table S1.

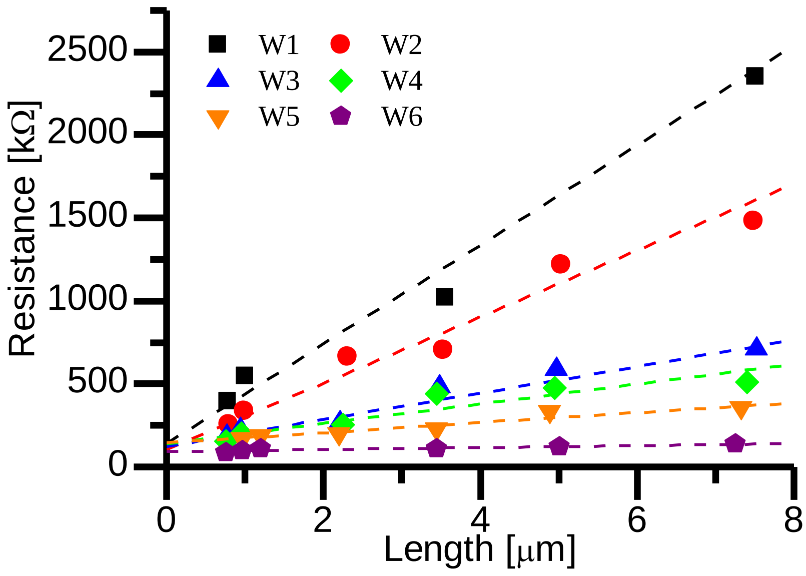

Figure S2. Extraction of the contact resistance. Total resistance vs. length of VO_2 devices before crystallization in the insulator phase.

Table S1. R_{con} for various VO_2 wire widths before crystallization in the insulator phase.

	***W*1**	***W*2**	***W*3**	***W*4**	***W*5**	***W*6**
**Dimension [μm]**	0.39	0.87	2.4	4.9	9.88	50
**R_{con} [kΩ]**	47.1	72.9	70.1	61.2	52.3	69.8
**Average [kΩ]**	**62.2**		**Standard Deviation [kΩ]**		**9.7**	

Similarly, R_{con} for the metallic phase before crystallization and R_{con} after crystallization in both phases were calculated. The values of R_{con} were used in the calculation of VO_2 resistivity (e.g., Figure 2c and 2d) and in the thermal simulations. To account for the change in contact resistance as a function of temperature over the phase transition in Figure 2d, we interpolated $R_{con}(T)$ using a lineshape function, $F(T)$, as follows:

$$R_{con}(T) = \left(R_{con,i} - R_{con,m}\right)[1 - F(T)] + R_{con,m}, \quad \text{(S1a)}$$

$$F(T) = \frac{1}{1 + e^{(343-T)\alpha}}, \quad \text{(S1b)}$$

where α = 0.6 is a unit-less parameter obtained from fitting *F(T)* to the measured total resistance, and $R_{con,i}$ and $R_{con,m}$ are respectively the insulator and metallic phases contact resistance. An interpolation procedure for the contact resistance was not applied for the resistance vs. current data due to the abrupt changes in the voltage. $R_{con,i}$ was used for the range between 0 mA and the first step in the transition, and $R_{con,m}$ was used for currents greater than that at the first transition.

With R_{con} known, the resistivity of the VO_2 wire (e.g., Figure 2c,d) was determined from

$$\rho_{VO2} = \frac{(R_{total} - 2R_{con})Wt}{L}, \quad \text{(S2)}$$

where *W* is the width, *t* is thickness, and *L* is the length of the VO_2 wire.

Crystallization in Wide VO_2 Wires

For wide (> 5 μm) VO_2 wires, the crystallized region did not cover the entire width of the wire. In Figure S3a (W = 10 μm), only the top region was smooth, and in Figure S3b (W = 50 μm), the crystallized region was only about 3 μm wide along the mid-section of the wire. Figure S4c and S3d display the *VI* characteristics before and after this partial crystallization. In Figure S4d, the "before" *VI* curve of a wide VO_2 wire shows only a single-step transition,[31] but the "after" *VI* curve exhibits the two-step transition characteristic of a narrow VO_2 wire. This suggests the current was channelled into a narrow, low resistivity VO_2 filament after the crystallization.

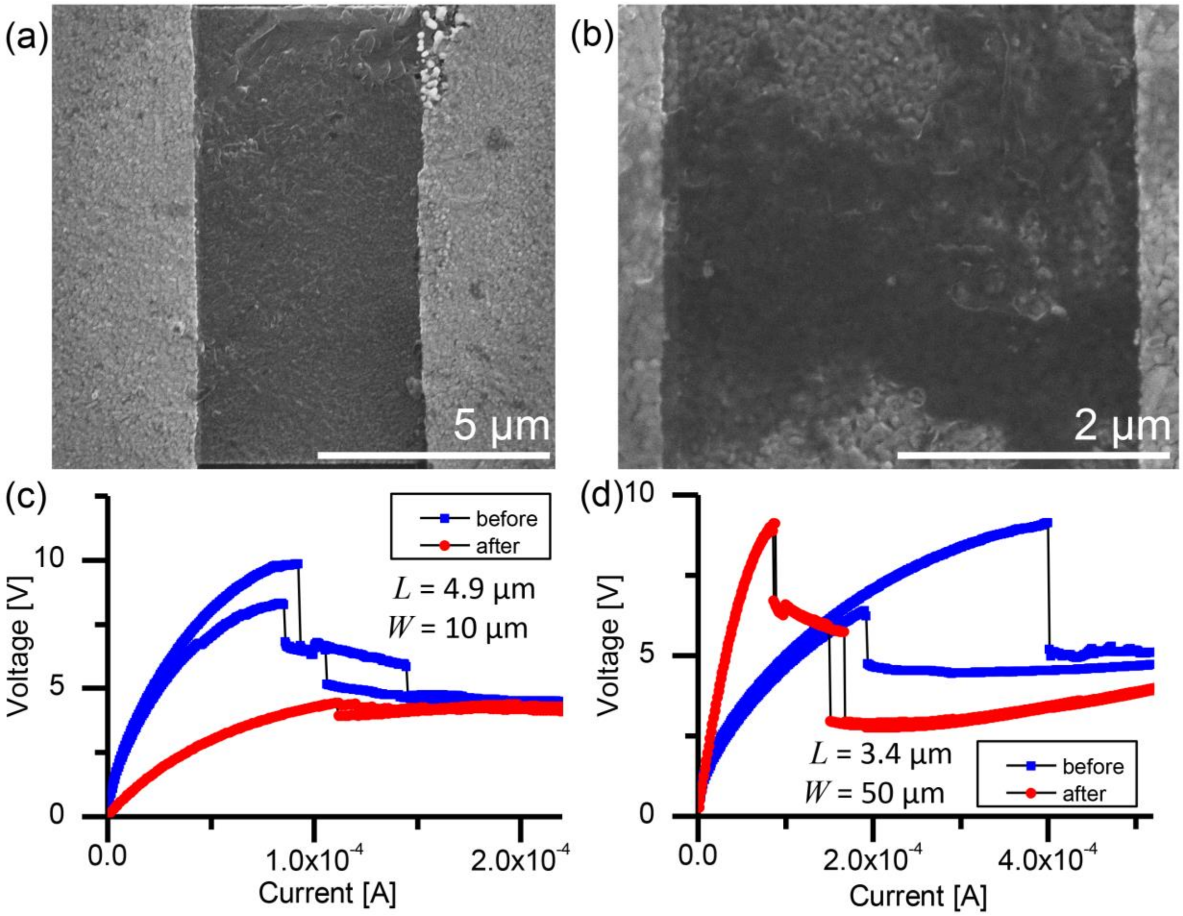

Figure S3. Partial crystallization in wide VO_2 wires. SEM image of VO_2 wires with (a) L = 4.9 μm and W = 10 μm and (b) L = 3.4 μm and W = 50 μm after I_c was applied. The VO_2 was not uniformly crystallized. The respective "before" and "after" *VI* plots are shown in (c) and (d).

X-ray Diffraction and Electron Microscopy

The X-ray diffraction was taken of the as-deposited VO_2 film using an X-ray diffractometer (Bruker D2 Phaser) with CuK_α radiation. The DIFFRAC.EVA V3.0 was used to calculate the weight percentage of VO_2 and V_2O_5.

For the TEM and SAED, the specimens were prepared using focused ion beam (FIB, Zeiss NVision 40). The FIB sectioned slices of 10×10 μm^2 samples with thicknesses between 50 nm to 75 nm of the as-deposited VO_2 film and crystallized VO_2 wire. TEM images and SAED patterns of the samples in the insulator and metallic phase were taken using a JEOL 2010F TEM/STEM with a temperature-controlled stage operated at 200 kV.

The electron diffraction patterns of the annealed VO_2 wire for both in the insulating phase and metallic phase are shown in Figure S4a and S4c, respectively (the metallic phase diffraction pattern is also shown in the main article). The computed diffraction patterns are simulated using SingleCrystal and the lattice parameters in ref 33. The results for the insulating (monoclinic) and metallic phase (rutile) are shown as black spots in Figure S4b and S4d, respectively. Only diffraction points with computed relative intensities >3.5% have been included. The faint spots near the centre of the diffraction patterns in Figure S4a and S4c are attributed to V_2O_5. In Figure S4b and S4d, we superimpose the computed diffraction pattern of V_2O_5 in red to find a good match between the relative position of the diffraction pattern of V_2O_5 to VO_2. The viewing directions of the VO_2 diffraction patterns are [112] and [210] in the monoclinic and rutiles phases, respectively, and the viewing direction of the V_2O_5 is $[5\bar{6}\bar{1}]$. The two VO_2 view directions are identical with respect to the sample. The different labelling of the crystal direction is due to the difference in the nomenclature of the *a* and *c* axes in the two phases.

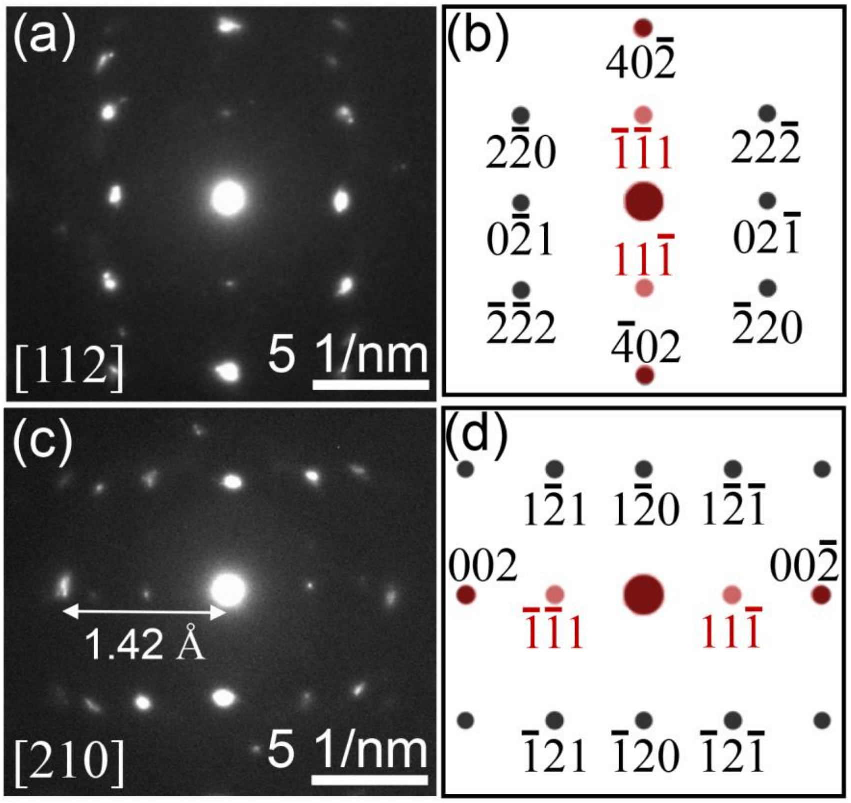

Figure S4. Measured and computed diffraction patterns. The electron diffraction pattern of crystallized VO_2 in the (a) insulating (monoclinic) and (b) metallic (rutile) phase and the respective computed diffraction pattern in the (b) and (d). The view directions used for the VO_2 diffraction calculations are given in (a) and (c). The red spots in (b) and (d) are from V_2O_5 at a view direction of $[5\bar{6}\bar{1}]$.
